# Trajectories of improvement with repetitive transcranial magnetic stimulation for treatment-resistant major depression in the BRIGhTMIND trial

**DOI:** 10.1038/s44184-024-00077-8

**Published:** 2024-06-27

**Authors:** P. M. Briley, L. Webster, S. Lankappa, S. Pszczolkowski, R. H. McAllister-Williams, P. F. Liddle, D. P. Auer, R. Morriss

**Affiliations:** 1 Mental Health and Clinical Neurosciences, School of Medicine, University of Nottingham, Nottingham, UK; 2 Nottingham National Institute for Health and Care Research (NIHR) Biomedical Research Centre, Nottingham, UK; 3 Nottinghamshire Healthcare NHS Foundation Trust, Nottingham, UK; 4 Sir Peter Mansfield Imaging Centre, School of Medicine, University of Nottingham, Nottingham, UK; 5 Translational and Clinical Research Institute and Northern Centre for Mood Disorders, Newcastle University, Newcastle upon Tyne, UK; 6 Cumbria, Northumberland, Tyne and Wear NHS Foundation Trust, Newcastle upon Tyne, UK; 7 NIHR Applied Research Collaboration East Midlands, University of Nottingham, Nottingham, UK; 8 NIHR Mental Health (MindTech) Health Technology Collaboration, University of Nottingham, Nottingham, UK

**Keywords:** Depression, Therapeutics

## Abstract

Repetitive transcranial magnetic stimulation (rTMS) is an established non-invasive brain stimulation treatment for major depressive disorder, but there is marked inter-individual variability in response. Using latent class growth analysis with session-by-session patient global impression ratings from the recently completed BRIGhTMIND trial, we identified five distinct classes of improvement trajectory during a 20-session treatment course. This included a substantial class of patients noticing delayed onset of improvement. Contrary to prior expectations, members of a class characterised by early and continued improvement showed greatest inter-session variability in stimulated location. By relating target locations and inter-session variability to a well-studied atlas, we estimated an average of 3.0 brain networks were stimulated across the treatment course in this group, compared to 1.1 in a group that reported symptom worsening (*p* < 0.001, *d* = 0.893). If confirmed, this would suggest that deliberate targeting of multiple brain networks could be beneficial to rTMS outcomes.

## Introduction

Repetitive transcranial magnetic stimulation (rTMS) delivers magnetic pulses over the scalp to alter activity in neural circuits. Usually delivered over left dorsolateral prefrontal cortex (DLPFC) in 20–30 daily sessions across 4–6 weeks, rTMS is a safe, and overall effective, treatment for major depressive disorder (MDD)^1,2^. However, it is known that there is large heterogeneity in clinical response between individuals^3,4^. Identifying different response patterns (trajectories) may help define depressive subtypes or facilitate decisions about when a treatment course should cease, or augmentation or an alternative stimulation approach be used.

Using group-based longitudinal trajectory modelling with weekly scores on an observer-rated measure of depressive symptoms (the Hamilton Depression Rating Scale, HDRS-17), collected as part of the largest randomised controlled trial of rTMS for depression so far (the THREE-D trial^5^), Kaster et al.^6^ distinguished four types of response trajectory: rapid-response (substantial improvement within the first week), two types of gradual (linear) response over 4–6 weeks, and non-response. However, there is evidence that a subpopulation of patients are late responders to TMS^7,8^, and delayed response has been described in antidepressant and psychotherapy studies^9,10^. It may be that weekly sampling of depression symptoms is too coarse to identify some trajectory types^11^. In addition, completion of scales such as the HDRS-17 takes around 20 min and involves a clinician interview, which is not always feasible in a busy clinical setting. If reliable and valid trajectories can be derived from a simple, easy-to-use, and quick-to-administer measure collected at each treatment session, outcome prediction from early response could be more readily incorporated into clinical practice.

BRIGhTMIND is a recently published double-blind randomised controlled trial of left DLPFC rTMS for treatment-resistant depression, comparing 20 sessions, over 4 weeks, of two rTMS pulse sequences and DLPFC localisation methods (ISRCTN registry #19674644)^12^. Specifically, it compared resting-state functional magnetic resonance imaging (rsfMRI) connectivity-guided intermittent theta burst stimulation (cgiTBS) and structural MRI neuronavigated rTMS delivered at the standard stimulation site (the “F3” location of the International 10-20 nomenclature, F3-rTMS). Standard symptom measures (including HDRS-17) were obtained at baseline, and 8-, 16- and 26-weeks following the commencement of treatment. None of these were collected during the treatment course. However, a simple self-rated measure—a five-item version of the patient global impression of change (PGIC)^13^, modified based on consultation with patient and public involvement advisers—was included at each daily treatment session. The modified PGIC (“mPGIC”) measure took just a few seconds. It asked people to rate, compared to before the start of the treatment course, whether they felt “much worse” (1), “a bit worse” (2), “just the same” (3), “a bit better” (4), or “much better” (5).

We used the data from the BRIGhTMIND trial in an exploratory analysis to identify whether distinct response trajectories could be identified from session-by-session mPGIC data. Beyond this, our objectives included:

1. Characterising distinct response trajectories over a course of 20 rTMS sessions in adults with treatment-resistant depression.
2. Validating response trajectories using other self- and observer-reported outcome variables at 8-, 16- and 26-weeks post TMS treatment commencement.
3. Exploring differences in clinical and treatment characteristics between response trajectories.

## Results

### Identifying the optimal trajectory model

**Fig. 1:**
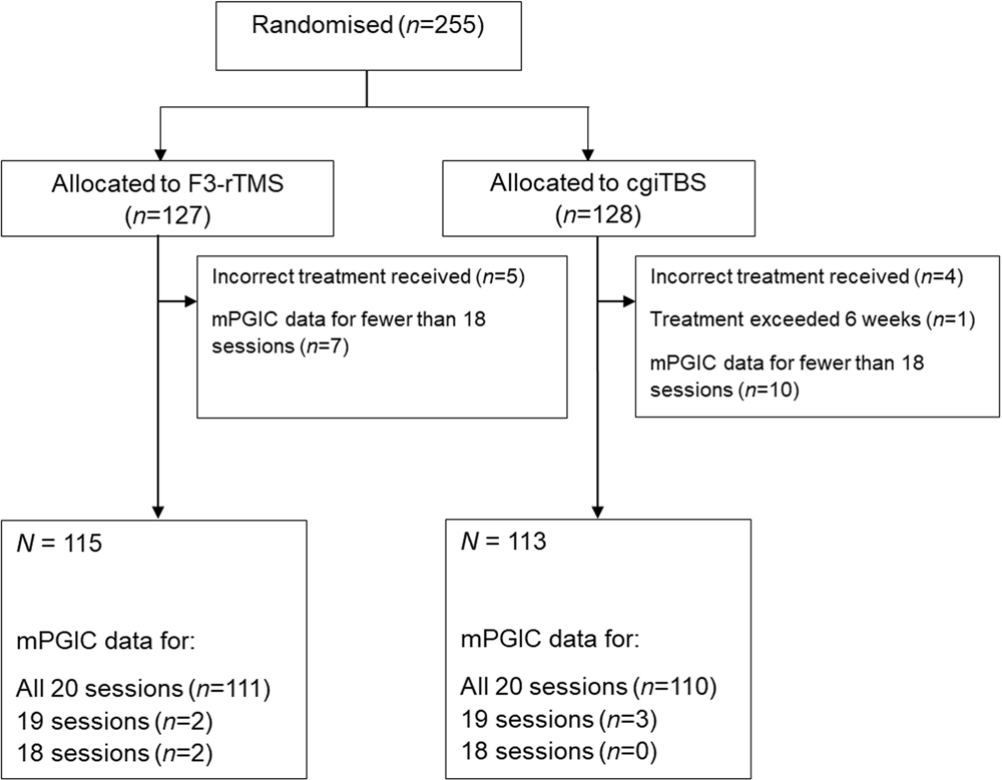
Datasets used for identifying response trajectories. Of the 255 patients included in the main trial analyses, 228 were included in the latent class analyses. Incorrect treatment received refers to cases in which stimulation was delivered to an unintended target (primarily cases where target co-ordinates were uploaded incorrectly to the neuro-navigation software).

Of the 255 patients included in the BRIGhTMIND intent-to-treat analysis^12^, nine were excluded from the current analysis due to receiving incorrect treatment and one due to their treatment period exceeding 6 weeks (Fig. 1). Seventeen patients were excluded due to having mPGIC data for fewer than 18 (90%) of the planned 20 sessions. The remaining data, for 228 participants, were entered into latent class growth analyses, fitting cubic response trajectories across daily mPGIC scores from the 20 stimulation sessions, for class sizes varying between one and eight. Bayesian Information Criterion (BIC, a measure of goodness of fit that includes a trade off with number of parameters—lower scores indicate better fit) decreased steeply across class sizes one to five and gradually after this, reaching a minimum at seven classes (Supplementary Fig. 1). Minimum class size was below the 5% threshold for the four-class (4.8%), seven-class (0.9%) and eight-class (4.8%) models, and the optimal six-class model did not achieve convergence. Therefore, we focus on the five-class model in the remainder of the report.

**Fig. 2:**
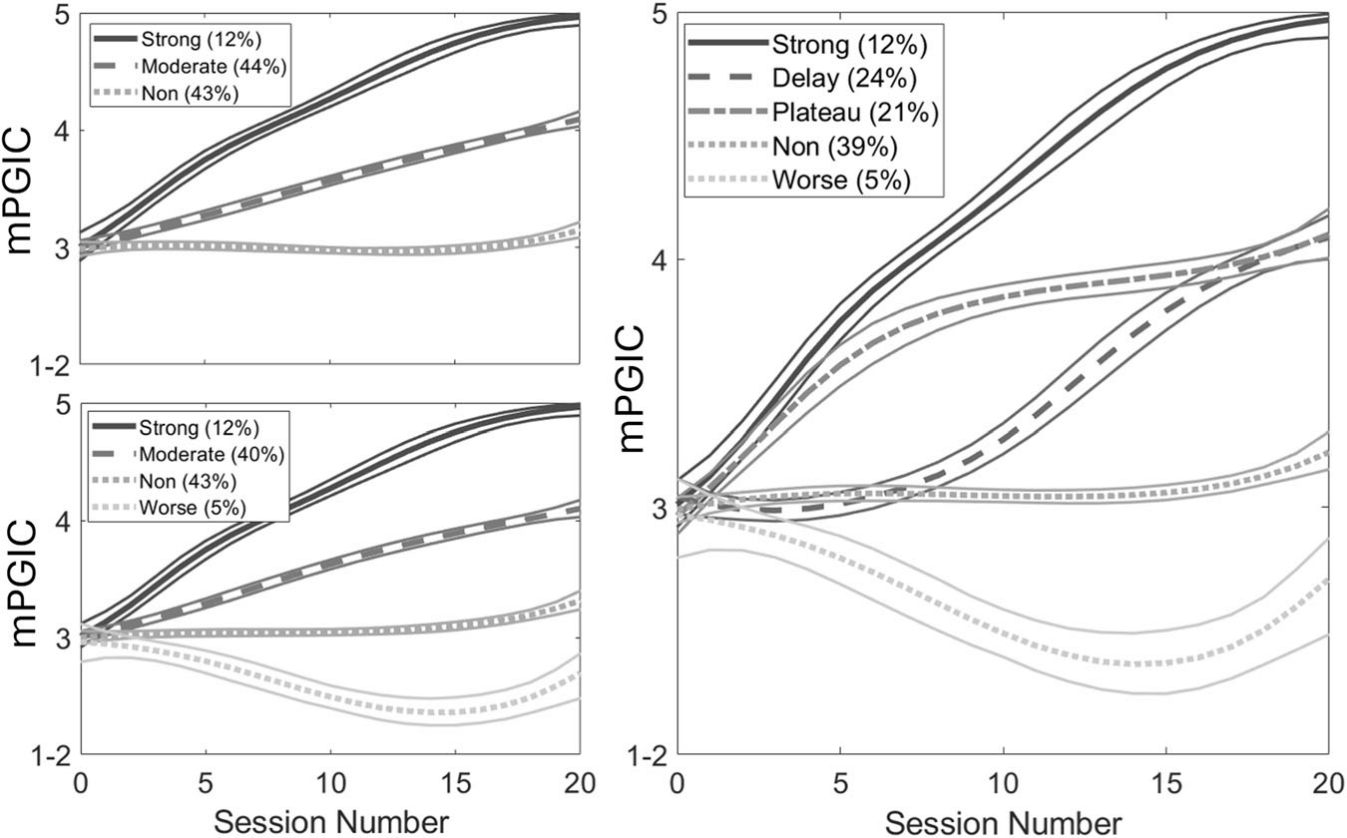
Predicted mPGIC trajectories for the three-, four- and five-class models. Includes 95% confidence bands computed from the posterior distributions of predicted values^42^. Percentages of the sample belonging to each class are given in the legends, along with class labels.

Figure 2 presents response trajectories for class sizes three to five (a flow chart indicating how members of each class are assigned in the next highest-class model is given in Supplementary Fig. 2). In summary, by the three-class model, substantial groups of non-improvers and moderate-improvers are identified, as well as a smaller group of patients that perceive strong early and continuing improvement leading to them feeling “much improved” overall. This “strong-improvement” group remains largely unchanged in the higher-class models. In the four-class model, the non-improvers are sub-divided into a larger group that report no change in difficulties and a smaller group that report worsening of difficulties during treatment. In the five-class model, the moderate-improvers are sub-divided into a group that shows early improvement that then plateaus, and a group that initially does not show improvement, but then begins to improve towards the end of week 2 (i.e., around session ten). It is notable that 10% of those classed as non-improvers by the four-class model are assigned to the delayed moderate-improver class in the five-class model. Latent class model fits to mPGIC scores from sessions 5, 10, 15 and 20 (mimicking weekly, as opposed to daily, temporal sampling of improvement) are presented in Supplementary Note 1 and model fits using days since treatment commencement, rather than session number, are presented in Supplementary Note 2.

### Five class model of response trajectories

We focus on the five-class model as this was the model with lowest BIC that met the minimum class size and convergence criteria. This model met a series of goodness-of-fit criteria^6,14,15^: average of maximum posterior probability of assignments (APPA, a measure of the probability of being assigned to the correct class) was 0.986, 0.932, 0.975, 0.986 and 0.959 for the strong improver, delayed moderate-improver, plateau moderate-improver, non-improver, and worsening classes respectively (values above 0.7 are typically regarded as acceptable^14^). Odds of correct classification (OCC, ratio of odds of correct classification—using maximum posterior probability classification rule—to the estimated proportions of class membership) was 515, 46, 143, 110 and 438 (values above 5 are acceptable^6^). Relative entropy was 0.951 (values above 0.8 are considered acceptable^15^).

### Comparison of outcomes at follow up between classes

**Fig. 3:**
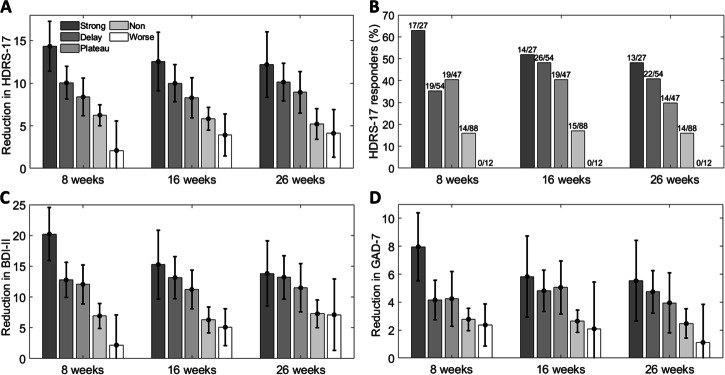
Improvement in symptoms across trajectory classes. Reduction from baseline in HDRS-17 (**A**), BDI-II (**C**), and GAD-7 (**D**) shown for each class at the 8-, 16- and 26-week follow-up time points (time points are measured relative to treatment commencement). Error bars are 95% confidence intervals. Percentage of HDRS-17 responders shown in (**B**) (values above bars are numbers of responders and total number in each class). Response defined as at least 50% reduction of total score from baseline. HDRS-17 Hamilton Depression Rating Scale, BDI-II Beck Depression Inventory, GAD-7 Generalised Anxiety Disorder Scale. Number of patients in each trajectory class are: Strong improvers (*n* = 27, 12% of sample), delayed improvers (*n* = 54, 24%), plateau improvers (*n* = 47, 21%), non-improvers (*n* = 88, 39%) and worsening (*n* = 12, 5%).

Overall, longer-term self- and observed-reported outcomes were superior in the strong improver group, then the delayed/plateau groups, then the non-improver group then the worsening group. At 8 weeks post-randomisation (approximately 4 weeks after the end of the treatment course), reduction in observer-reported depressive symptoms (HDRS-17) was significantly greater in all improver groups than in the worsening group (Fig. 3A). HDRS-17 reduction was also significantly greater in the strong- and delayed-improver groups than in the non-improver group, and in the strong-improver group than in the delayed-improver and plateau groups. These effects were largely sustained at 16- and 26-weeks post-randomisation. It is notable that some degree of improvement was observed even in the non-improver group. However, none of the participants in the “worsening” class were HDRS-17 responders (at least 50% score reduction) at any time point (Fig. 3B and Supplementary Fig. 3). Similar patterns were seen for self-reported depressive (BDI-II, Fig. 3C; PHQ-9, Supplementary Fig. 4A) and anxiety symptoms (GAD-7, Fig. 3D), as well as social and occupational functioning (WSAS, Supplementary Fig. 4B).

### Comparison of treatment parameters between classes

**Fig. 4:**
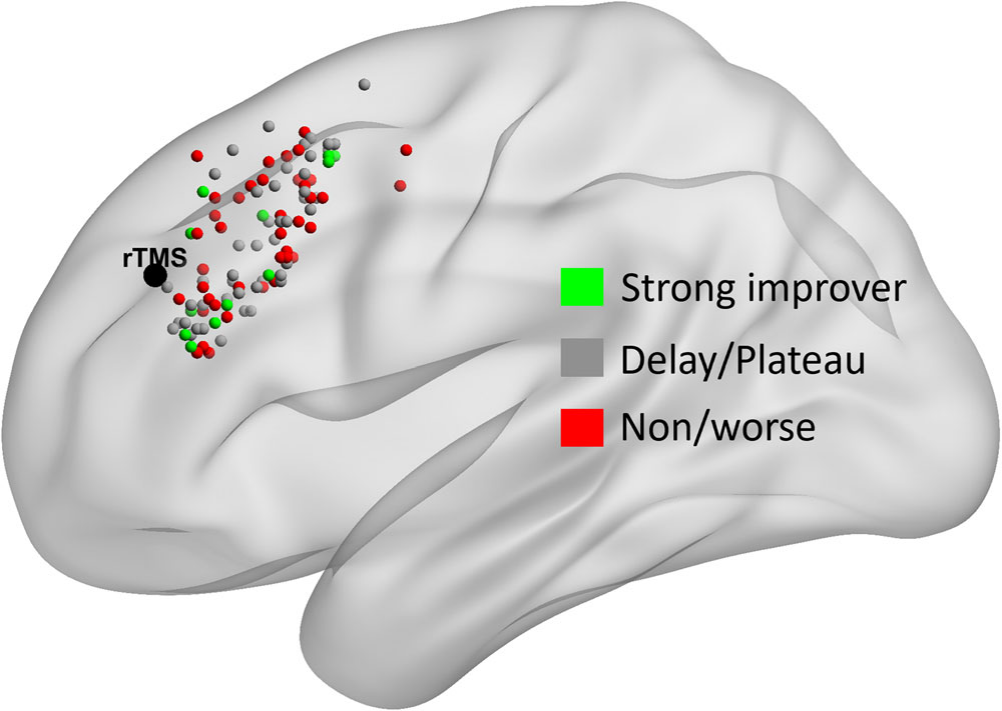
Stimulation target locations for each trajectory class. Target locations are shown as spheres for each patient that received cgiTBS (for ease of visualisation, the two moderate-improver groups are both coloured grey and the non-improver/worsening groups are both coloured red). The target for those that received rTMS is also shown (black). Plotted on a smoothed ICBM152 brain using BrainNet Viewer^45^.

Comparisons of treatment parameters across trajectory classes are shown in Table 1 (comparisons of demographic and clinical parameters are presented in Supplementary Note 3 and the predictive value of these parameters alongside treatment parameters is presented in Supplementary Note 4). There were no significant differences between classes in: target location (for those that received cgiTBS, as the target remained the same for those that received F3-rTMS, Fig. 4); mean distance of stimulation from the intended target; or mean distance of stimulation from the location used in the first session. However, *standard deviation* of distance from session one across sessions did differ significantly across classes. Specifically, standard deviation (representing between-session variability in stimulation location) was greater in the strong-improver group (4.9°) than the delayed-moderate improver (3.3°, n.s.), plateau-moderate improver (2.9°, *p* = 0.044, *d* = 0.461), non-improver (2.8°, *p* = 0.023, *d* = 0.406) and worsening (2.1°, *p* = 0.002, *d* = 0.521) groups. Significant relationships between standard deviation and reduction in HDRS-17 were also seen in the whole sample for each follow-up time point (*r* = 0.295/0.257/0.260, *p* < 0.001, for 8-, 16-, and 26-weeks; these reduced to *r* = 0.141/0.123/0.196, *p* = 0.063/0.105/0.013, when the strong-improver group was excluded). There was a similar trend across trajectory classes using standard deviation of location *within* a session (Table 1).

**Table 1:** Comparison of treatment variables across classes of the five-class model

	Class 1 Strong (*n* = 27)	Class 2 Delayed-Mod (*n* = 54)	Class 3 Plateau-Mod (*n* = 47)	Class 4 Non-impr. (*n* = 88)	Class 5 Worse (*n* = 12)	Tests of differences
Medication change	No 81.5% Yes 18.5%	75.9% 24.1%	78.7% 21.3%	76.1% 23.9%	83.3% 16.7%	*χ*^2^(4) = 0.691, *p* = 0.952
Treatment adjusted	No 70.4% Yes 29.6%	85.2% 14.8%	66.0% 34.0%	67.0% 33.0%	83.3% 16.7%	*χ*^2^(4) = 7.424, *p* = 0.115
Intensity (percent)	55.5 (50.6–60.4)	55.5 (52.0–59.0)	50.7 (46.9–54.5)	51.6 (48.9–54.4)	53.5 (46.2–60.8)	*F*(4,215) = 1.374, *p* = 0.244
(cgiTBS group) Mean target co-ordinate (MNI)	−42/25/33	−40/22/36	−41/23/34	−41/21/37	−43/22/38	*V*(Pillai’s Trace) = 0.052, *F*(12,312) = 0.455, *p* = 0.939
Mean dist. from target^a^ (mm)	7.0 (4.8–9.7)	5.8 (4.4–7.5)	5.7 (4.2–7.6)	5.1 (4.1–6.3)	3.9 (1.9–6.9)	*F*(4,215) = 0.946, *p* = 0.438
Mean dist. from sess. 1^a^ (mm)	7.6 (5.7–9.9)	6.4 (5.2–7.9)	6.8 (5.4–8.5)	5.6 (4.7–6.6)	4.8 (2.9–7.5)	*F*(4,213) = 1.325, *p* = 0.262
SD of dist. from sess. 1^a^ (mm)	4.9 (3.7–6.4)^3–5^	3.3 (2.6–4.1)	2.9 (2.2–3.8)^1^	2.8 (2.3–3.3)^1^	2.1 (1.2–3.6)^1^	*F*(4,213) = 3.215, *p* = 0.014*, *η*_p_^2^ = 0.057
Networks stimulated	3.0 (2.3–3.6)^3–5^	2.3 (1.8–2.7)^5^	1.7 (1.2–2.2)^1^	1.6 (1.3–2.0)^1^	1.1 (0.1–2.0)^1,2^	*F*(4,205) = 4.716, *p* = 0.001**, *η*_p_^2^ = 0.084
Within-session SD of dist. (mm)^a^	2.3 (1.7–3.1)	1.8 (1.4–2.2)	1.9 (1.5–2.4)	1.6 (1.3–1.9)	1.1 (0.6–1.9)	*F*(4,213) = 2.080, *p* = 0.084^^^, *η*_p_^2^ = 0.038
(cgiTBS group) Target distance from −44/40/29^a^	19.1 (14.4–24.8)	22.4 (18.5–26.7)	19.6 (16.3–23.4)	23.4 (20.3–26.8)	21.1 (14.2–29.9)	*F*(4,105) = 0.863, *p* = 0.489

For binary variables, values are percentage of patients. For continuous variables, values are means with 95% confidence intervals. Tests of significance of differences across classes are given in the final column (^^^*p* < 0.1, *p < 0.05, **p < 0.01). For significant variables, superscript numbers denote pairs of classes with significant pairwise differences at the *p* < 0.05 level (superscripts correspond to class numbers, such that a superscript of 4 indicates a significant difference from class 4).

^a^Statistical analyses performed on the cube root of values due to right skew of values across patients (presented values are back transformed).

### Networks stimulated across trajectory classes

The brain is organised into networks of co-operating regions that can be identified at rest or during tasks from inter-region correlations of the fMRI signal^16^. We examined whether differences in inter-session location variability might equate to differences in the number of DLPFC-connected brain networks stimulated during a treatment course. For each patient, we extracted their target location and the locations of four hundred points within a sphere, centred at the target location, with radius equal to twice the standard deviation of stimulation location across sessions. For each of these locations, we identified the nearest node (smallest Euclidean distance) in Power et al.^17^ resting-state-network atlas and extracted the network label assigned by Power et al. to that node. We then calculated the number of unique network labels across nodes for that participant.

Number of networks stimulated (unique network labels) was significantly greater for the strong improver group (3.0) than the plateau (1.7, *p* = 0.017, *d* = 0.718), non-improver (1.6, *p* = 0.012, *d* = 0.830) and worsening (1.1, *p* < 0.001) groups, and greater for the delayed improver (2.3) than the worsening group (*p* < 0.001, *d* = 0.893). That is, on average, three distinct networks were stimulated in the strong improver group, but only one network in the worsening group. Percentage of patients for whom this approach suggests that more than one network was stimulated was: 63.0% (strong improver), 40.4% (delayed improver), 38.1% (plateau), 37.7% (non-improver) and 8.3% (worsening). For the strong improver group, the Executive Control Network (ECN), Default Mode Network (DMN), and Ventral Attention Network (VAN) were stimulated in 96.3, 59.3, and 44.4% of patients. These values were 97.4, 36.4, and 18.2% for the non-improver group, and 91.7, 16.7 and 0.0% for the worsening group. Comparison across trajectories in right anterior insula connectivity, and target distance to a previously suggested optimal site, are presented in Supplementary Note 5.

## Discussion

We derived trajectories of improvement to 20 sessions of left DLPFC rTMS in people with treatment-resistant major depressive disorder, using a single-item self-report measure collected at each treatment session. The use of a quick and simple measure, with high sampling frequency (each session), are unique features of this rTMS study. A five-class model of perceived improvement fit the data well, and included “strong improvers”—noticing some improvement within the first week and feeling much improved by the end of the treatment course; “delayed improvers”—not noticing benefit till halfway through the course, then improving till treatment end; “plateau improvers”—noticing initial improvement but failing to improve further; non-improvers—not noticing improvement; and a small group who reported worsening across sessions. Location of stimulation was similar across trajectories, but, surprisingly, strong improvers had significantly more session-to-session variability in stimulated location than other classes.

Our trajectories of strong, moderate, and non-improvers are largely consistent with those identified for trials of rTMS^6,18^, medications^19,20^, psychotherapy^9^ and transcranial direct current stimulation^21^. To our knowledge, this is the first study to identify a delayed improvement trajectory to rTMS. A prior psychotherapy trial, which also assessed response daily, identified a delayed-response class alongside classes of no/limited response, early subtle response, and early rapid response^9^. A study examining weekly change in HDRS-17 found a class of delayed (>2 week) responders in a combined sample of patients receiving psychotherapy or antidepressants, but not when examining each therapeutic option separately^10^. An antidepressant study examining weekly change in HDRS-17 and using no minimum class size threshold, extracted a nine-class solution including delayed response classes^22^. Our exploratory latent class growth analysis using sessions 5, 10, 15 and 20 only (mimicking weekly symptom assessments) selected a model incorporating only a single class of moderate improvers, alongside strong and non-improvers (Supplementary Note 1). It was possible to derive noisier trajectories that sub-divided moderate improvers to include a delayed-improver class by removing the minimum class size threshold. We suggest that examining improvement at the session-by-session level best captures the delayed-improver trajectory, by adequately sampling the change in symptoms over time. Approaches that enable even higher temporal sampling of symptoms (such as electronic diaries or wearable devices that derive metrics of mood from movement^23^) may be able to further enhance trajectory analyses.

In a large rTMS registry study, Hutton et al.^24^ found that weekly self-reported (PHQ-9) depression scores, averaged across patients, continued to improve beyond 20 or 30 sessions. We propose three potential mechanisms for how this translates into improvement at the individual level: (1) Expected additional improvement in the delayed-improver group; (2) Additional improvement in strong improvers that cannot be detected by the mPGIC (see later); (3) Emergence of a second class of delayed improvers beyond 20 sessions. Disentangling these possibilities will be an important next step. Finally, the non-improver group (although much less so the worsening group) did show some post-treatment improvement in self- and observer-rated measures. This could reflect spontaneous improvement in depression (although patients in the BRIGhTMIND trial typically had long duration of current illness and a high degree of treatment resistance). Alternatively, it could reflect improvement that initially goes unrecognised or unacknowledged by the patient themselves, or a delayed effect of rTMS that emerges post-treatment.

Unexpectedly, our strong improvers had greater across-session variability in stimulated location than other classes. Whilst variability in stimulated location was relatively small due to the use of neuro-navigation in the BRIGhTMIND trial, we showed that the greater variability would be expected to be associated with stimulation of a greater number of DLPFC-connected brain networks. This suggests that the precision targeting used in BRIGhTMIND had a counterintuitive negative effect (as random co-targeting of additional networks was associated with better outcomes). Alternatively, it is possible that precision targeting introduced an unexpected bias away from a single optimal site. Arguing against the latter, we did not observe spatial clustering of targets for patients with similar response trajectories. Also, in those that received cgiTBS, target proximity to a previously proposed optimal stimulation site (based on connectivity with the subgenual anterior cingulate cortex)^25^ did not differ between groups.

We showed that the greater inter-session location variability in strong improvers would particularly be associated with additional stimulation of the “ventral attention network” (involved in identifying the most important stimuli in the internal and external worlds) and the “default mode network” (involved in internal-world processing and rumination)^16^. Studies motivating the BRIGhTMIND trial highlighted the importance of target connectivity with the right anterior insula (predominantly involved in the VAN) for mediating treatment response^26–28^, and other studies have identified crucial roles for the DMN^29^, leading to exploration of alternative target sites that are more closely involved in the DMN (particularly, dorsomedial prefrontal cortex^30,31^). An open question is around the origin of the inter-session location variability itself. The variability could be random, due to incidental coil placement inaccuracies, or it could be systematic, with patients with higher location variability moving more during a session. Further work is needed to disentangle these possibilities.

The trajectory classes we identify, as well as relationships between trajectory and treatment (or clinical) factors, should be regarded as exploratory and require confirmation in future studies. We argue for the validity of a five-class model in our dataset but decisions on class number are dependent on modelling assumptions and selection criteria. For example, Kaster et al. allowed different trajectories classes to have different polynomial degree (linear, quadratic, or cubic). Facilitated by our higher temporal sampling, we chose to use only cubic trajectories as these would capture the full range of expected response patterns and not require a trade-off between number of classes and polynomial degree. Supplementary Fig. 2 shows the relationships between different class models in our sample. We suggest that these largely represent similar distinctions at different granularities. Our self-report mPGIC measure is limited by a patient’s recent experience of living with depression. That is, whilst patients in the strong-improver group might feel “much improved” compared to baseline (a mPGIC of 5) by around session ten, their context for this judgement is the past months or years lived with depression. Feeling much improved does not preclude further perceived improvement with additional sessions. It should be noted that our five-item mPGIC outcome measure was derived from the more commonly used seven-item PGIC^13^, based on feedback from our patient and public involvement advisers prior to study commencement. The aim was to reflect language that patients themselves would use to describe change in their depression—“improved” was replaced with “better”, “minimally” with “a bit”, and “no change” became “just the same”. Whilst we were able to derive meaningful trajectories from this simple measure, which related to established self- and observer-rated scales, the reliability and validity of the mPGIC has not been independently demonstrated. Finally, in the BRIGhTMIND trial, for ethical and clinical reasons, patients who reported feeling the same or worsening during the treatment course were reviewed at 16/26 weeks (8 weeks if suicide risk was identified) by a psychiatrist with additional expertise in mood disorders. This could result in recommendations for further treatment being passed to the health professional that referred them into the study. Nevertheless, the proportion of patients with medication or psychological treatment changes between baseline and the 16-week follow-up was similar across trajectory classes (Supplementary Table 1).

The present study demonstrates that distinct trajectories of improvement to rTMS can be derived from a simple, self-report, measure, with validity supported by relationships to other, well-established, self-report and observer-rated measures, and that a substantial proportion of patients notice delayed improvement during a treatment course. The strong-improver class had higher variability in inter-session treatment location. It may be that such variability allows stimulation of non-ECN DLPFC-connected brain networks with important roles in the pathophysiology of depression (particularly DMN and VAN). While preliminary, this observation, together with the lack of clinical benefit from the cgiTBS approach used in the BRIGhTMIND trial, calls for a conceptual re-evaluation of precision targeting of non-invasive brain stimulation treatment for depression. It may be that deliberate targeting of multiple brain networks, or further optimisation of stimulation protocols through acceleration and synchronisation, are needed, warranting more in-depth mechanistic investigations.

## Methods

### BRIGhTMIND trial

Between January 2019 and January 2022, 255 participants were randomised to the BRIGhTMIND trial (*n* = 128 cgiTBS, *n* = 127 F3-rTMS). ISRCTN registry (ISRCTN19674644). The trial was approved by the East Midlands Leicester Central Research Ethics Committee (no. 18/EM/0232). The planned analyses for the trial have been recently reported^12^. Briefly, eligible participants were aged ≥18 years, had current treatment-resistant unipolar major depressive disorder (≥2 Massachusetts General Hospital Staging Score^32^ which was adapted for new treatment options), with depression rated as moderate to severe (≥16 on the 17-item GRID Hamilton Depression Rating Scale, GRID-HDRS-17^33^). Participants were scheduled to receive 20 sessions over 4-6 weeks of cgiTBS or F3-rTMS. All aspects of the design, conduct, analysis, and interpretation of the main trial were assisted by a Lived Experience Advisory Panel of people who have experienced severe depression, some of whom also had experience of rTMS.

Participants assigned to cgiTBS received 50 Hz bursts of three pulses (80% resting motor threshold), with bursts repeating every 200 ms (5 Hz). Bursts were presented in 10-s cycles, consisting of 2 s of stimulation and 8 s of rest. There were 20 such cycles per run (600 pulses/run). Five runs were presented per session, with 5-min inter-run intervals (3000 pulses/session). The cgiTBS brain target was determined using a baseline resting-state fMRI scan and defined based on Granger Causality Analysis as the location within left DLPFC receiving maximal effective connectivity from the right anterior insula (MNI co-ordinates: x = 30, y = 24, z = −14 mm)^34^.

Participants assigned to F3-rTMS followed the standard US Food and Drug Administration approved protocol^35^. Stimulation was at 120% motor threshold with 75 4 s trains of 10 Hz interspersed by 26 s intertrain intervals, with a total of 3000 pulses per session. The F3-rTMS brain target was determined using the participants’ structural MRI so as to target a standard MNI co-ordinate x = −41, y = 43, z = 32 mm (selected a priori as the parenchymal voxel closest to the “F3” site in a standard brain).

During each treatment session, stimulation location was directed using the StimGuide Navigated TMS Package (Magstim Co.). Stimulation location could be adjusted prior to the start of each session due to discomfort, keeping within 2 cm of the target site. Participants were advised to keep their head still during stimulation. The system monitored and stored coil location relative to the head throughout a treatment session.

### Inclusion criteria for current analyses

Participants were excluded from the current trajectory analyses if (1) mPGIC data were missing for more than two treatment sessions (i.e., more than 10% of the total planned sessions, *N* = 17); (2) treatment was delivered at an unintended site (this arose due to mistakes in the uploading of co-ordinates to the targeting system during the trial, *N* = 9); (3) there was greater than 6 weeks between the first and final treatment sessions (*N* = 1). The latter two criteria are the same as those used in the pre-specified imaging analysis protocol. The included sample (*N* = 228) contained 113 patients who received cgiTBS and 115 who received F3-rTMS. Most (*N* = 221) had mPGIC data for 20 sessions, five had data for 19 sessions and two for 18 sessions. Missing sessions were entered into the analyses as missing values. Comparisons of clinical and treatment variables across trajectory classes used all available data.

### Assessments and measures

Patients completed a shortened version of the patient global impression of change measure (PGIC^13^) at the end of every TMS session. They were asked to report change relative to the pre-treatment baseline. The modified measure (“mPGIC”) was on a 5-point Likert scale (1 = much worse, 2 = somewhat worse, 3 = just the same, 4 = somewhat better, 5 = much better). We used the mPGIC data to derive response trajectories/classes.

To determine the validity of the classes derived from the mPGIC measures, we compared classes on change in other outcome measures that were collected at baseline and 8, 16, and 26 weeks after randomisation. For this, we used the continuous outcome data from the GRID-HDRS-17, Beck Depression Inventory-II (BDI-II^36^), Patient Health Questionnaire (PHQ-9^37^), Work and Social Adjustment Scale (WSAS^38^), and the Generalised Anxiety Disorder Assessment (GAD-7^39^), as well as categorical outcome data calculated from the GRID-HDRS-17 by splitting participants into responders (≥50% reduction in score) and non-responders.

To determine whether there are baseline clinical or demographic factors that predict response trajectory, we compared trajectory classes on those variables that were examined as moderators of the primary outcome in the main BRIGhTMIND report^12^. These included baseline GRID-HDRS-17^33^, GAD-7^39^, MGH-S^32^, age, gender, childhood trauma questionnaire (CTQ^40^) and treatment arm. Kaster et al.^6^ also examined baseline benzodiazepine use as a predictor of response classes. However, in our sample, fewer than 10% of patients (*n* = 21 of 228) were taking anxiolytics or hypnotic medication at baseline, so we did not include this as a comparison variable. To understand further findings around depression severity and co-morbidities, we compared trajectory classes on proportions of patients taking an antidepressant or antipsychotic medication. Trial data were also available for mood stabilisers and stimulants, but these were taken by fewer than 10% of our sample so were not examined further (*n* = 16 and 4, respectively, out of 228).

We compared trajectory classes on proportion of patients with co-morbid anxiety disorders (generalised anxiety disorder, social anxiety disorder, panic disorder or agoraphobia), co-morbid eating disorders (anorexia or bulimia nervosa, binge eating disorder) and with psychotic features (hallucinations or delusions, judged as “mood congruent” or “mood incongruent”)—all assessed at the trial baseline SCID-5-RV interview (Supplementary Note 3). Proportion of patients with co-morbid obsessive compulsive disorder (OCD) was available from the trial, but not examined here as OCD was present in fewer than 10% of patients (*n* = 18 of 228).

To determine whether treatment factors predicted response trajectory, we compared trajectory classes on two nominal variables (whether treatment had to be adjusted for tolerability according to the trial protocol—intensity reduced or location site adjusted up to 2 cm; whether patients had medication changes during the treatment course) and seven continuous variables (mean stimulation intensity across sessions, mean Euclidean distance from planned target, mean Euclidean distance from the location delivered in session one and standard deviation of this measure, mean within-session standard deviation in location, and, for the patient who received cgiTBS, mean target co-ordinate and target distance from a previously suggested optimal site). Due to marked right skew in residuals, the cubic root was taken prior to statistical analyses for the distance variables. The reported means and 95% confidence intervals for these variables in Table 1 were calculated on the cubic root values, then transformed back by taking the cube.

We also used target location and between-session standard deviation in stimulated location to estimate the number of DLPFC-connected brain networks stimulated during the treatment course for each patient. For each patient, we identified the nearest node (smallest Euclidean distance in MNI space) from Power et al.^17^ resting-state atlas to the target location and to 400 additional points within a sphere centred at the target location with radius corresponding to twice the standard deviation of stimulated location across sessions. Specifically, the 400 points lay on the surface of 20 spheres with radii varying from one tenth to twice the standard deviation of stimulation location (20 points on the surface of each sphere). The atlas network-label assigned to each node by Power et al. was extracted. Number of unique labels was taken as a measure of the number of networks stimulated by treatment. We also examined the percentage of patients in each class who would have (according to the above approach) had stimulation of the Executive Control Network (ECN; grouping together the “fronto-parietal task control network” and “dorsal attention network” atlas labels), Default Mode Network (DMN; “default mode” label), and Ventral Attention Network (VAN; “salience”, “cingulo-opercular task control” or “ventral attention” labels).

### Deriving trajectory classes

All analyses was completed in R (version 4.3.1^41^), using the Latent Class Mixed Model toolbox (LCMM version 2.0.2^42^), with additional fit indices calculated with the Latent Class Trajectory Modelling Tools toolbox (LCTMtools^14^). In each case, a baseline mPGIC value, for session zero, was set at 3 (i.e., no change from baseline). Given the rarity of mPGIC value one (much worse), being used by only 3% of participants and being present in fewer than 1% of data points, values of one and two were combined to create a four-value ordinal scale. The *lcmm* function was used with the “thresholds” link function, which is designed for ordinal outcome variables. All models used cubic polynomials to account for mPGIC change across sessions, as cubic polynomials are able to describe the range of expected trajectories (including delayed improvement). The single-class model was fitted first, followed by models with classes two-through-eight. A maximum of one thousand iterations was allowed per fit. One hundred starting values were used for each of the multi-class models, informed by parameters from the one-class model, using the *gridsearch* function, with the best-performing model serving to represent that class number. We initially ran models with treatment group (cgiTBS or F3-rTMS) in the class membership model. As this predictor was non-significant in all cases, models were re-run without this term.

Model selection used the Bayesian Information Criterion (BIC^43^)—lower values indicating better fit^44^, the minimum class size—it has been recommended that models containing classes comprising less than 5% of the total sample should not be retained^15^, and convergence of fit (using default LCMM criteria, i.e., convB, convL, and convG = 0.0001). These metrics were also used by Kaster et al.^6^. For the chosen model, fit indices were calculated to confirm adequacy^14,15^, as detailed in the main text.

Following this, using the chosen model, we compared clinical and treatment variables between participants from different classes, using chi-square tests and independent-sample ANOVAs as appropriate (for cgiTBS treatment location, a MANOVA was used with x-, y- and z co-ordinate in MNI space as dependent variables). We also compared classes on outcomes at the 8-, 16-, and 26-week follow-up time points separately (note that time is measured relative to the treatment start). All statistical tests were performed in IBM SPSS Statistics (v29.0.1.0), were two-tailed, with alpha set to 0.05. For pair-wise *t*-tests, equal variance was assumed, unless Levene’s test for equality of variances was significant.

### Supplementary information

Supplemental information

## Data Availability

Analysis code and mPGIC values used for deriving response trajectories (alongside fitted latent class models) are available at: https://github.com/pmbriley/trajectories. Demographic, clinical outcome, and treatment variables used in the current study will be made available on the University of Nottingham data repository (https://rdmc.nottingham.ac.uk).

## Code availability

Analysis code used for deriving response trajectories (and fitted latent class models) are available at: https://github.com/pmbriley/trajectories.
