# Supplementary material for: Trajectories of improvement with repetitive transcranial magnetic stimulation for treatment-resistant major depression in the BRIGhTMIND trial
Source: Npj Ment Health Res. 2024 Jun 27;3:32. doi: 10.1038/s44184-024-00077-8 (PMC11211415; doi:10.1038/s44184-024-00077-8)
Supplement: Supplementary file 1 — Supplemental information [file 44184_2024_77_MOESM1_ESM.pdf]

SUPPLEMENTAL INFORMATION

Trajectories of improvement with repetitive transcranial magnetic stimulation for treatment-resistant major depression in the BRIGhTMIND trial

P. M. Briley, L. Webster, S. Lankappa, S. Pszczolkowski, R. H. McAllister-Williams, P. F. Liddle, D. P. Auer, R. Morriss

Supplemental Note 1

Trajectories of improvement using weekly, as opposed to daily, temporal sampling

To determine whether the fine (daily) temporal sampling of perceived symptom change was necessary for identifying the five trajectory classes, we also fitted latent class mixed models to weekly (i.e., days five, ten, fifteen and twenty) mPGIC scores. Minimum BIC was found for the six-class model, but minimum class size dropped below the 5% threshold from the four-class model onwards. The three-class model resembled that derived from the full dataset (see main text Fig. 2), with strong- (14% of total sample), moderate- (56%), and non-improver (30%), trajectories, although separation between classes was poorer (relative entropy of 0.845, versus 0.972 for the three-class model fitted to the full dataset) and the "moderate" class contained a substantial number of patients classed as non-improvers in the full-dataset model (Supplemental Fig. 5). In the four-class model, a small (3.5%) worsening class emerged, and in the five-class model, differentiation of moderate improvers into plateau (31%) and delayed (15%) moderate improvers was evident, again with poorer separation between classes (relative entropies of 0.862 and 0.822 for four- and five-class, versus 0.967 and 0.951 for the corresponding full dataset models). Therefore, it is possible to derive the five trajectory classes using sparser temporal sampling, although the resulting classes show poorer separation (and the trajectories have wider confidence bands) than when trajectories are derived from daily sampling.

Supplemental Note 2

Trajectories of improvement using days since treatment commencement in place of session number

Supplemental Figure 6 shows trajectories of improvement for models fitted using "days since start of treatment" as the independent variable, in place of session number.

Trajectories are similar to those in Figure 2 of the main text. Considering the five-class model, relative entropy is a little lower (0.940), with slightly poorer fit metrics for the delayed-, plateau-, and non-improver classes (APPA: 0.923 versus 0.932, 0.937 versus 0.975, 0.983 versus 0.986), and slightly better metrics for the strong-improver and worsening classes (APPA: 0.993 versus 0.986, 0.985 versus 0.959). Whilst consistent with the main analyses, understanding the extent to which TMS effects accumulate across time versus across stimulation sessions, within a treatment course, could lead to more efficient or more acceptable treatment regimens. This could be examined in a future study by, for example, comparing trajectories of improvement between patients receiving daily, versus alternate-daily, stimulation.

Supplemental Note 3

Comparison of baseline demographic and clinical features between classes

Demographic and baseline variables for each class are shown in Supplemental Table 1. There were no significant differences in the proportion of patients receiving F3-rTMS or cgiTBS within each group (in addition, the treatment type term in the original class-membership model was non-significant for each class). Consistent with Kaster et al.[1], our worsening group had highest baseline depression severity, although differences were small. Baseline HDRS-17 was significantly lower for the plateau-improver (21.9) than either the delayed- (24.4, p=0.011, Cohen's d=0.520) or worsening (25.8, p<0.001, d=0.964) classes. Presence of psychotic features at baseline (hallucinations or delusions, as assessed with the SCID-5-RV interview) differed across classes – proportion of patients with psychotic features was significantly greater in the strong-improver class (33.3%) than the plateau (10.6%, p=0.016), non-improver (11.4%, p=0.007), and worsening (0.0%, p=0.023) classes. A similar pattern was seen when examining presence of mood congruent (typical depressive themes of guilt, nihilism, deserved punishment or personal inadequacy) or mood incongruent (non-typical themes) psychotic features separately, although neither sub-variable alone was significant. An early review of predictors of improvement with rTMS suggested that psychotic features were associated with *poorer* response to rTMS[2], although psychotic features have featured little in subsequent reviews and have often been an exclusion criterion in rTMS trials. Presence of psychotic features is a predictor of good response to electroconvulsive therapy for MDD[3,4].

Proportion taking an antidepressant differed across classes – being significantly greater for all improver categories (≈85%) than the worsening class (41.7%, p=0.001-0.005), and for the delayed-improver (85.2%) than non-improver class (70.5%, p=0.046). A higher response rate in patients taking antidepressants was found in an analysis of data from eleven rTMS trials[5]. Proportion taking an antipsychotic also differed across classes – it was significantly less in the strong-improver group (3.7%) than the delayed- (24.1%, p=0.022) or non-improver (21.6%, p=0.032) group (although no patients in the worsening group were taking an antipsychotic). Concurrent antipsychotic use has previously been associated with poorer response to rTMS in MDD[6].

Supplemental Note 4

Clinical and treatment factors entered together to predict trajectory class

To understand the relative importance of significant clinical (baseline HDRS-17, presence of psychotic features, current antidepressant medication, current antipsychotic medication) and treatment predictors of trajectory class (number of networks stimulated), we entered the variables together into a multinomial logistic regression to predict trajectory class (Supplemental Table 2). Non-improver was set as the reference class. Initially, the worsening class was omitted due to no variation in presence of psychotic features or current antipsychotic use for this group (to enable the model to be accurately fitted). All predictors were significant, apart from baseline HDRS-17. Significant predictors of the strong-improver class were: greater number of networks stimulated [B = 0.451, $W(1)$ = 9.864, p = 0.002], presence of psychotic features [B = 1.926, $W(1)$ = 9.530, p = 0.002], absence of antipsychotic medication [B = 2.197, $W(1)$ = 4.105, p = 0.043], and taking an antidepressant [B = 1.302, $W(1)$ = 3.906, p = 0.048]. Significant predictors of the delayed-improver class were: greater number of networks stimulated [B = 0.282, $W(1)$ = 4.653, p = 0.031] and taking an antidepressant [B = 0.969, $W(1)$ = 4.086, p = 0.043]. There were no significant predictors of the plateau class. Repeating the analysis, but omitting psychotic features and current antipsychotic use, and including the worsening class, baseline HDRS-17 was significant. Otherwise, there was no change to the pattern of the above findings for the included variables. Significant predictors of the worsening class were: greater baseline HDRS-17 [B = 0.144, $W(1)$ = 4.118, p = 0.042] and absence of antidepressant medication [B = 1.623, $W(1)$ = 5.467, p = 0.019].

Supplemental Note 5

Differences in right anterior insula connectivity and distance from target to a previously proposed optimal stimulation site

For patients that received cgiTBS (as the intended target varied in these patients), we compared the magnitude of the distance from the intended stimulation target to a previously-reported optimal stimulation target at MNI co-ordinates 44/40/29 (calculated to maximise group-level anti-correlation with the subgenual anterior cingulate cortex, sgACC[7]). No significant difference across trajectory classes was found (p = 0.489). Finally, given that baseline right anterior insula (rAI) "net outflow" – the balance of influence between rAI and left DLPFC, calculated using effective connectivity analyses – predicted symptom improvement in the main trial analyses[8], for which it was a pre-specified analysis, we examined how this metric varied across classes (see Supplemental Figure 7).

Supplemental Figure 1

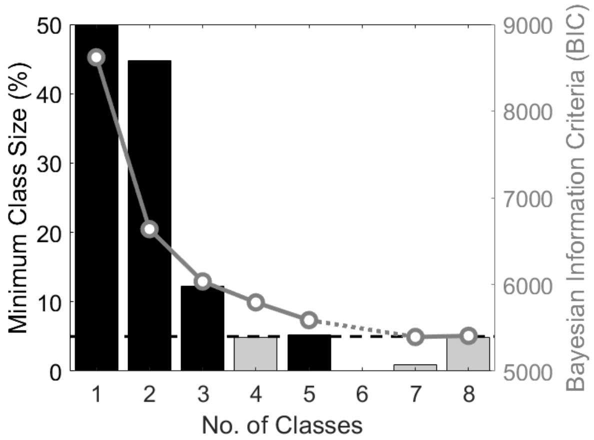

Selecting the optimal number of latent class trajectories. *Bayesian Information Criteria (BIC, circles) and minimum class size (percent of total sample, bars) for models with class sizes one to eight. The 5% minimum class size threshold is shown as a black horizontal dashed line. Bars for models that did not meet this threshold are shaded grey. Data points for the six-class model are omitted as this model did not meet convergence criteria.*

Supplemental Figure 2

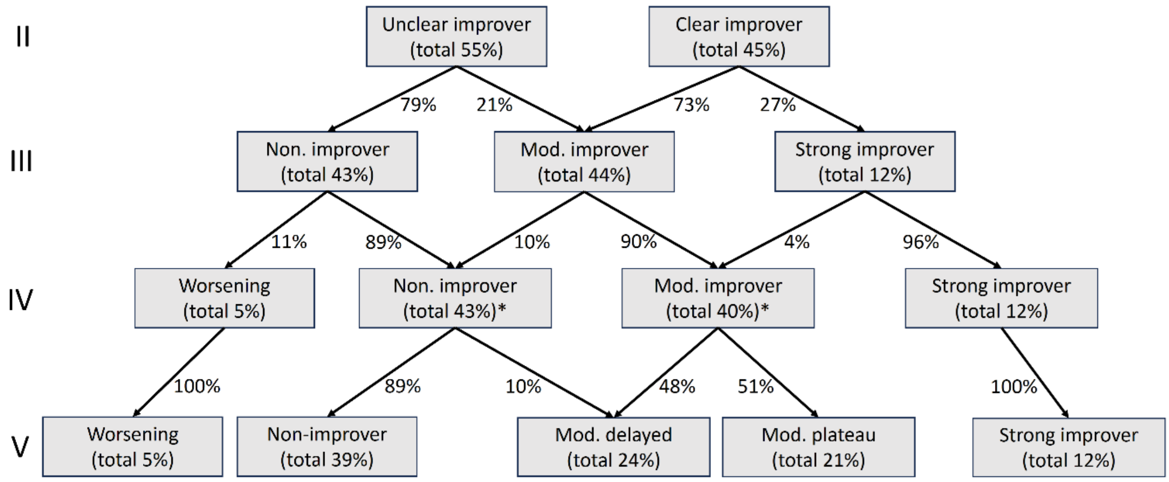

Assignment of participants to trajectory classes across models. *Each box gives a class label and percentage of participants assigned to that class. Arrows show the percentage of participants from a lower-class model assigned to a class in a higher-class model. 1% of participants in the four-class moderate-improver class were assigned to the five-class non-improver class; 1% of participants in the four-class non-improver class were assigned to the five-class worsening class (these instances are indicated by asterisks).*

Supplemental Figure 3

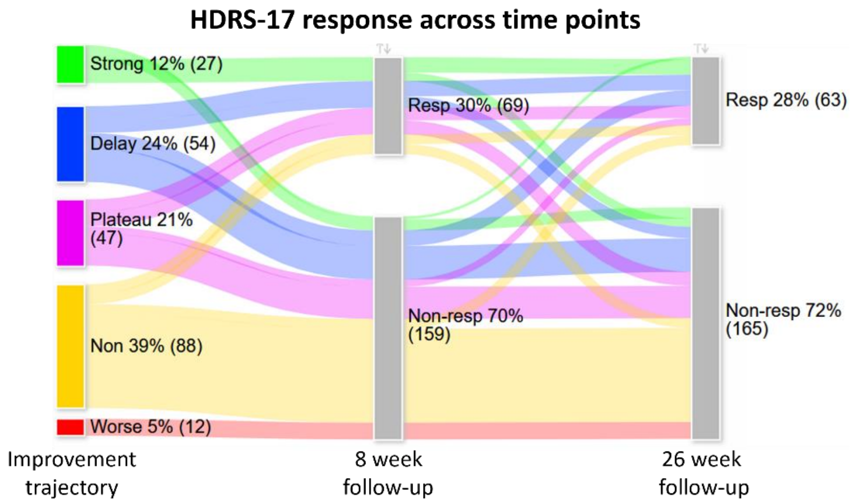

Sankey diagram illustrating proportion of patients in each trajectory class who are 8- and 26-week responders (resp) and non-responders (non-resp) on the HDRS-17. *Response defined as at least a 50% reduction in HDRS-17 total score from baseline. Plotted with ChartExpo (v1.0.0.7, PolyVista).*

Supplemental Figure 4

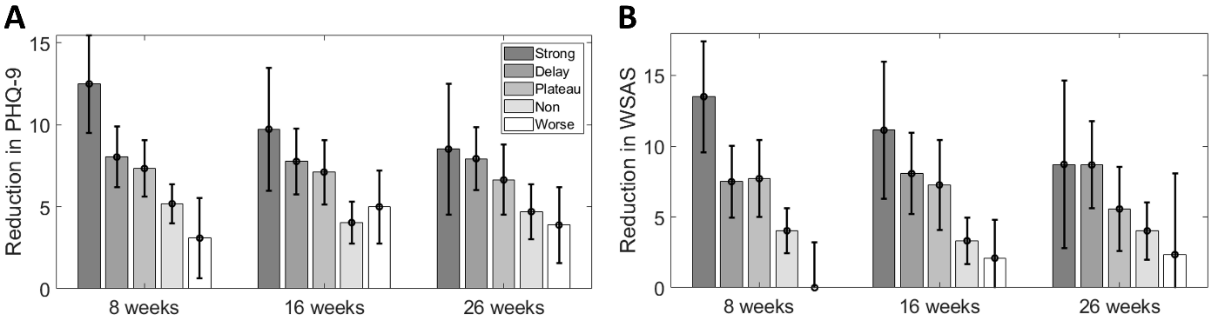

Improvement in symptoms and functioning across trajectory classes. *Shown for reduction in Patient Health Questionnaire (PHQ-9, A) and reduction in Work and Social Adjustment Scale (WSAS, B). Error bars are 95% confidence intervals.*

Supplemental Figure 5

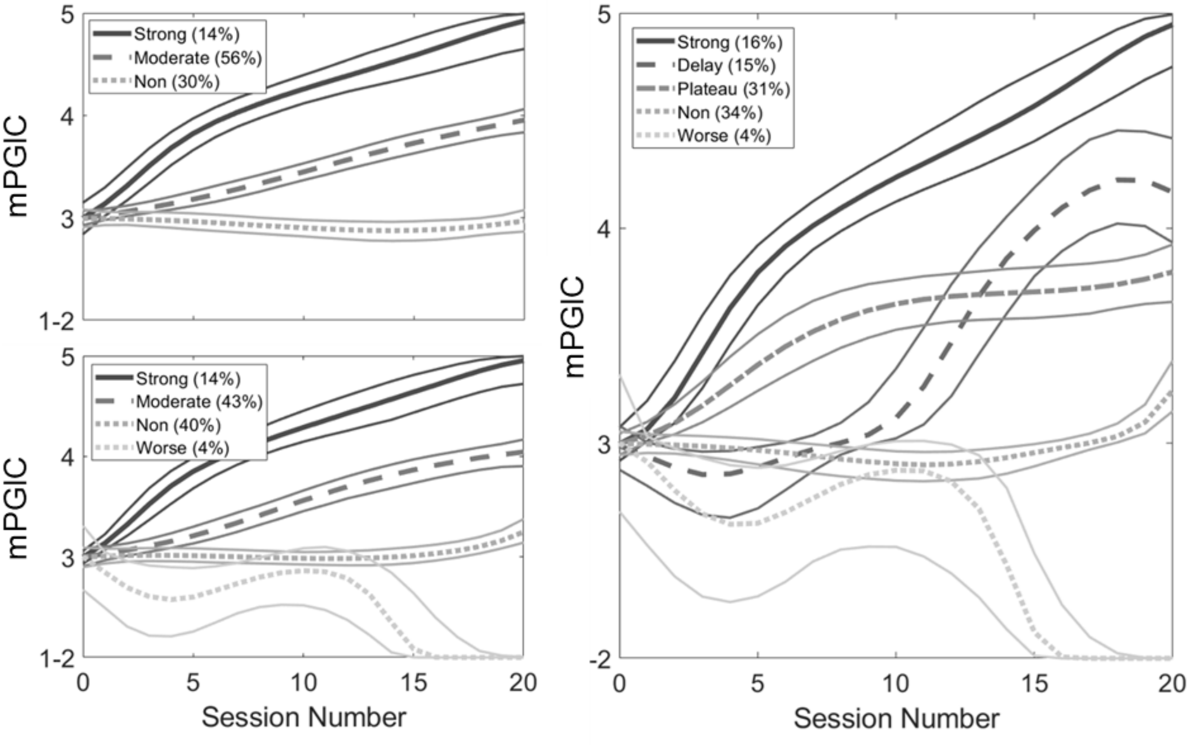

Predicted trajectories for models fitted to weekly mPGIC scores. *Includes 95% confidence bands computed from the posterior distributions of predicted values*[9]*. Percentages of the sample belonging to each class are given in the legends, along with class labels.*

Supplemental Figure 6

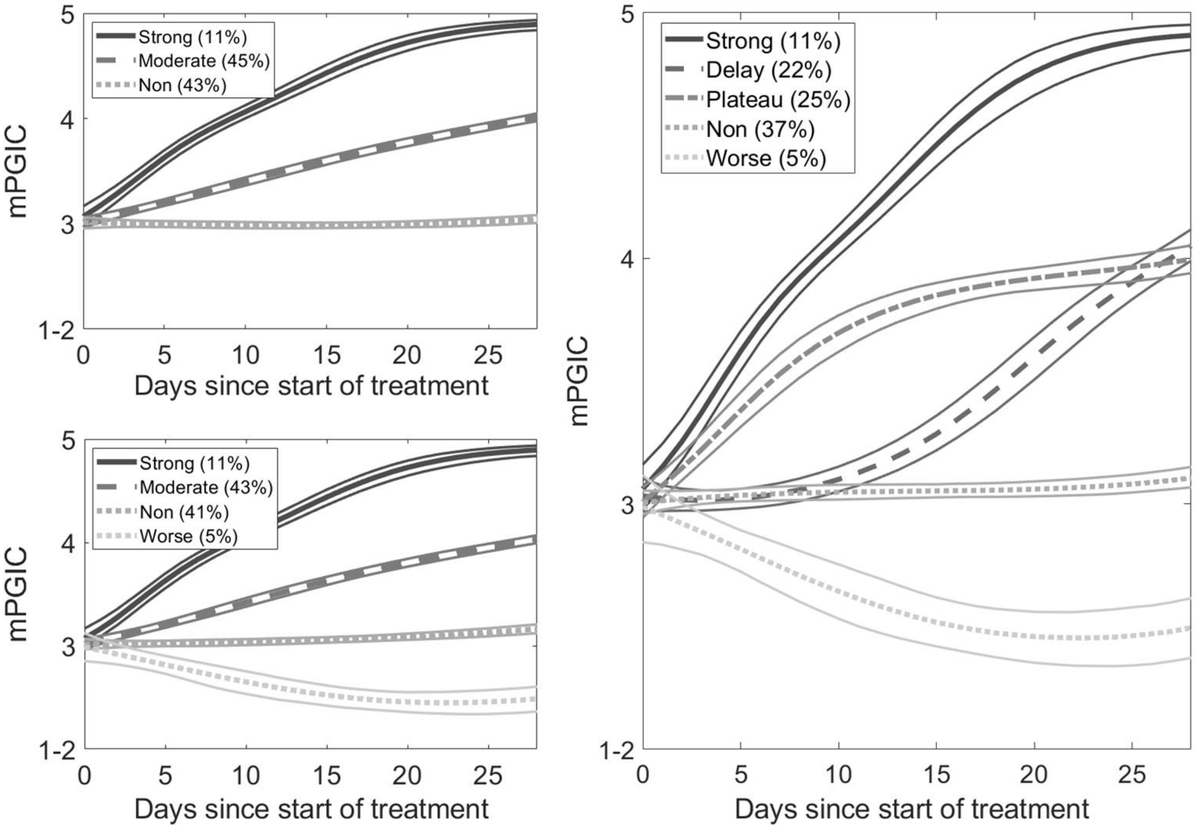

Predicted trajectories for models fitted using "days since start of treatment" in place of "session number" as the independent variable. *Includes 95% confidence bands computed from the posterior distributions of predicted values*[9]*. Percentages of the sample belonging to each class are given in the legends, along with class labels.*

Supplemental Figure 7

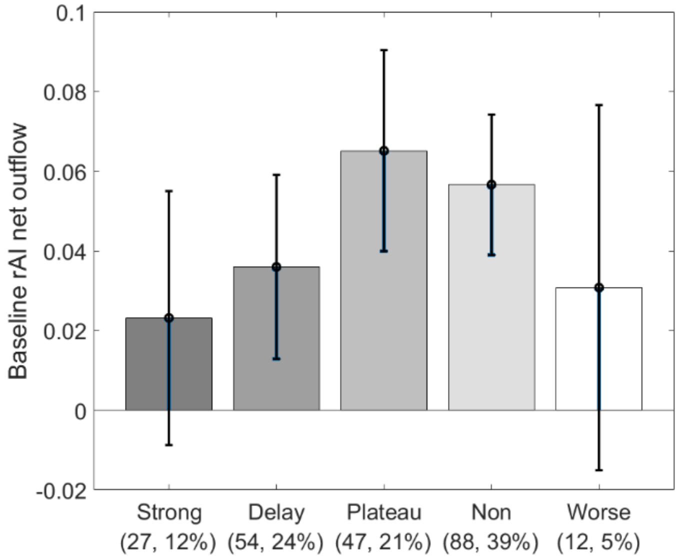

Baseline right anterior insula net outflow across trajectory classes. *Right anterior insula (rAI) "net outflow" to left dorsolateral prefrontal cortex (DLPFC). Net outflow – the balance of influence between regions – calculated as effective connectivity from rAI to DLPFC minus effective connectivity from DLPFC to rAI, as in Morriss et al.*[8]*. Error bars are 95% confidence intervals. Values below each axis label give number and percentage of participants in each trajectory group. The net outflow estimate for the worsening group was imprecise. The strong improvers showed low net outflow (in line with the main trial results), whilst the non-improvers showed higher net outflow and the delayed improvers were intermediate. The plateau group showed similar net outflow to the non-improvers.*

Supplemental Table 1

	Class 1 Strong (*n* = 27)	Class 2 Delayed-Mod (*n* = 54)	Class 3 Plateau-Mod (*n* = 47)	Class 4 Non-impr. (*n* = 88)	Class 5 Worse (*n* = 12)	Tests of differences
Female	59.3%	53.7%	44.7%	53.4%	50.0%	$\chi2(4)=1.728$, $p=0.786$
Male	40.7%	46.3%	55.3%	46.6%	50.0%	
F3-rTMS	55.6%	55.6%	42.6%	50.0%	50.0%	$\chi2(4)=2.025$, $p=0.731$
cgiTBS	44.4%	44.4%	57.4%	50.0%	50.0%	
rMT	55.9% (54.6-57.2)	56.1% (54.8-57.4)	54.5% (53.2-55.9)	53.5% (52.3-54.7)	54.1% (53.2-55.0)	$F(4,212)=0.733$, $p=0.570$
MGH-S	5.5 (4.1-6.9)	5.8 (4.9-6.8)	5.0 (4.0-6.1)	6.8 (6.0-7.5)	5.7 (3.7-7.8)	$F(4,223)=1.995$, $p=0.096$^, $\eta_p^2=0.035$
Age	42.7 (37.2-48.1)	46.0 (42.1-49.8)	43.2 (39.1-47.3)	44.9 (41.9-48.0)	42.4 (34.3-50.6)	$F(4,223)=0.428$, $p=0.788$
CTQ total	52.5 (45.9-59.1)	46.5 (41.9-51.2)	47.0 (42.0-52.1)	44.0 (40.3-47.7)	39.3 (29.1-49.4)	$F(4,210)=1.706$, $p=0.150$
B/L HDRS-17	23.6 (21.8-25.4)	24.4 (23.1-25.7)[3]	21.9 (20.6-23.3)[2,5]	23.5 (22.5-24.5)	25.8 (23.0-28.5)[3]	$F(4,223)=2.448$, $p=0.047$*, $\eta_p^2=0.042$
B/L GAD-7	13.6 (11.9-15.4)	13.0 (11.8-14.3)	13.1 (11.8-14.5)	13.1 (12.1-14.1)	14.5 (11.9-17.1)	$F(4,223)=0.316$, $p=0.867$
Psychotic features	33.3%[3-5]	22.2%	10.6%[1]	11.4%[1]	0.0%[1]	$\chi2(4)=12.415$, $p=0.015$*
- mood congruent	18.5%	16.7%	4.3%	6.8%	0.0%	$\chi2(4)=9.146$, $p=0.058$^
- mood incongruent	14.8%	5.6%	6.4%	4.5%	0.0%	$\chi2(4)=4.735$, $p=0.316$
Melancholic features	70.8%	88.7%	77.8%	77.9%	100.0%	$\chi2(4)=7.268$, $p=0.122$
Atypical features	20.8%	5.7%	8.9%	9.3%	0.0%	$\chi2(4)=5.966$, $p=0.202$
Taking antidepress.	85.2%[5]	85.2%[4,5]	85.1%[5]	70.5%[2]	41.7%[1,2,3]	$\chi2(4)=15.483$, $p=0.004$**
Taking antipsychot.	3.7%[2,4]	24.1%[1]	12.8%	21.6%[1]	0.0%	$\chi2(4)=9.619$, $p=0.047$*
Co-morbid anxiety disorder	74.1%	77.8%	74.5%	77.3%	58.3%	$\chi2(4)=2.265$, $p=0.687$
Co-morbid eating disorder	29.6%	18.5%	14.9%	20.5%	8.3%	$\chi2(4)=3.459$, $p=0.484$

Comparisons of baseline demographic and clinical variables across classes of the five-class model. *For binary variables, values are percentage of patients. For continuous variables, values are means with 95% confidence intervals. Tests of significance of differences across classes given in final column (Chi-square for nominal variables and ANOVA for continuous variables; ^ = $p<0.1$, * = $p<0.05$, ** = $p<0.01$). For variables where overall Chi-square or ANOVA was significant ($p<0.05$), superscript numbers denote pairs of classes with significant pair-wise differences ($p<0.05$ using proportion- or t-test) – superscripts correspond to class numbers, such that a superscript of 4 indicates a significant difference from class 4. MGH-S: Massachusetts General Hospital Staging model for treatment-resistant depression, CTQ: childhood trauma questionnaire, B/L HDRS-17: Baseline Hamilton Depression Rating Scale, B/L GAD-7: Baseline Generalised Anxiety Disorder questionnaire; co-morbid anxiety disorder (presence of generalised anxiety disorder, social anxiety disorder, panic disorder or agoraphobia at baseline, diagnosed with SCID-5-RV); OCD: obsessive compulsive disorder; rMT: resting motor threshold.*

Supplemental Table 2

	**Class 1 Strong (*n* = 27)**	**Class 2 Delayed-Mod (*n* = 54)**	**Class 3 Plateau-Mod (*n* = 47)**	**Class 4 Non-impr. (*n* = 88)**	**Class 5 Worse (*n* = 12)**
B/L HDRS-17	*B = -0.05* (-0.16- 0.06) *p*=0.347	*B = 0.01* (-0.06- 0.09) *p*=0.736	*B = -0.07* (-0.16-0.01) *p*=0.094^	Reference class	*B = 0.14* (0.00-0.28) *p*=0.042*
Psychotic features	*B = 1.93* (0.70-3.15) *p*=0.002**	*B = 0.970* (-0.08-2.02) *p*=0.069^	*B = 0.48* (-0.76-1.72) *p*=0.449	Reference class	n.a.
Taking antidepress.	*B* = 1.30 (0.01-2.59) *p*=0.048*	*B* = 0.97 (0.03-1.91) *p*=0.043*	*B = 0.94* (-0.04-1.91) *p*=0.059^	Reference class	*B = -1.62* (-2.98- -0.26) *p*=0.019*
Taking antipsychot.	*B* = -2.20 (-4.32- -0.07) *p*=0.043*	*B = -0.10* (-1.00-0.81) *p*=0.832	*B = -0.82* (-1.94-0.30) *p*=0.153	Reference class	n.a.
Networks stimulated	*B* = 0.45 (0.17-0.73) *p*=0.002**	*B* = 0.28 (0.03-0.54) *p*=0.031*	*B = 0.08* (-0.25-0.41) *p*=0.644	Reference class	*B = -1.73* (-3.61-0.14) *p*=0.070^

Significant individual predictors of trajectory class, entered together into multinomial logistic regressions. *Values (B) are change in log odds due to a one unit increase in each predictor (with 95% confidence interval). Non-improvers served as the reference class. ^p<0.1, * p<0.05, ** p<0.01. Values for classes 1-3 are from an analysis incorporating all five predictors but excluding the worsening class. Values for the worsening class (class 5) are from an analysis incorporating all classes but excluding the psychotic features and antipsychotic medication predictors (due to no variation in these predictors in the worsening class).*

Supplementary References

1. Kaster, T. S. *et al.* Trajectories of response to dorsolateral prefrontal rTMS in major depression: A THREE-D study. *American Journal of Psychiatry* **176**, 367-375 (2019).
2. Gershon, A. A., Dannon, P. N. & Grunhaus, L. Transcranial Magnetic Stimulation in the Treatment of Depression. *American Journal of Psychiatry* **160**, 835–845 (2003).
3. Pinna, M. *et al.* Clinical and biological predictors of response to electroconvulsive therapy (ECT): a review. *Neuroscience Letters* 669, 32-42 (2018).
4. Haq, A. U., Sitzmann, A. F., Goldman, M. L., Maixner, D. F. & Mickey, B. J. Response of depression to electroconvulsive therapy: A meta-analysis of clinical predictors. *Journal of Clinical Psychiatry* **76**, 1374-1384 (2015).
5. Fitzgerald, P. B., Hoy, K. E., Anderson, R. J. & Daskalakis, Z. J. A study of the pattern of response to rTMS treatment in depression. *Depress Anxiety* **33**, 746-753 (2016).
6. Hebel, T., Abdelnaim, M., Deppe, M., Langguth, B. & Schecklmann, M. Attenuation of antidepressive effects of transcranial magnetic stimulation in patients whose medication includes drugs for psychosis. *Journal of Psychopharmacology* **34**, 1119-1124 (2020).
7. Fox, M. D., Buckner, R. L., White, M. P., Greicius, M. D. & Pascual-Leone, A. Efficacy of transcranial magnetic stimulation targets for depression is related to intrinsic functional connectivity with the subgenual cingulate. *Biol Psychiatry* **72**, 595–603 (2012).
8. Morriss, R. *et al.* Connectivity-guided intermittent theta burst versus repetitive transcranial magnetic stimulation for treatment-resistant depression: a randomized controlled trial. *Nat Med* **30**, 403-413 (2024).
9. Proust-Lima, C., Philipps, V. & Liquet, B. Extended Mixed Models Using Latent Classes and Latent Processes [R package lcmm version 2.0.2]. *J Stat Softw* **78**, (2023).
